# Decision support tools for pancreatic cancer detection: external validation in Australian primary care — a retrospective cohort study

**DOI:** 10.3399/BJGP.2025.0328

**Published:** 2026-02-24

**Authors:** Silja Schrader, Meena Rafiq, Javiera Martinez-Gutierrez, Mary Waterhouse, Belinda Lee, Rachel E Neale, Jon Emery

**Affiliations:** 1 Centre for Cancer Research and Department of General Practice and Primary Care, University of Melbourne, Melbourne, Australia; 2 Faculty of Medicine, Dentistry and Health Sciences, University of Melbourne, Melbourne, Australia; 3 Epidemiology of Cancer and Healthcare outcomes (ECHO) group, UCL, London, UK; 4 Population Health Program, QIMR Berghofer, Brisbane, Australia; 5 Division of Systems Biology and Personalised Medicine, Walter and Eliza Hall Institute of Medical Research, Parkville, Victoria, Australia; 6 Department of Medical Oncology, Peter MacCallum Cancer Centre, Melbourne, Victoria, Australia; 7 Department of Medical Oncology, The Northern Hospital, Epping, Victoria, Australia; 8 School of Public Health, The University of Queensland, Brisbane, Australia; 9 Population and Global Health, Lee Kong Chian School of Medicine, Nanyang Technological University, Singapore, Singapore

**Keywords:** general practice, pancreatic cancer, risk assessment tools, decision support tools, primary health care, cohort studies

## Abstract

**Background:**

Pancreatic cancer is often diagnosed at an advanced stage with poor survival. Risk assessment tools have been developed to aid early diagnosis of pancreatic cancer in primary care settings (QCancer^®^, electronic Risk Assessment Tool [eRAT], and the Queensland Institute of Medical Research [QIMR] Berghofer Pancreatic Cancer Decision Support Tool [QPaC Tool]) but have not been validated in the Australian setting.

**Aim:**

To estimate and compare the performance of these tools for identifying patients with undiagnosed pancreatic cancer using Australian primary care data.

**Design and setting:**

A cohort study was conducted using linked primary care and cancer registry data from Victoria, Australia.

**Method:**

Patients presenting to primary care with signs and/or symptoms included in the tools (recorded in the primary care ‘reason for encounter’) were included. Diagnostic accuracy statistics for each tool (and their individual signs and symptoms) were compared.

**Results:**

Patients with pancreatic cancer were more likely (*P*<0.001) to present with new-onset diabetes, jaundice, and unexpected weight loss pre-diagnosis than patients without pancreatic cancer. The most common pre-diagnostic presentations in patients with pancreatic cancer were jaundice (29.0%), abdominal pain (25.6%), change in bowel habits (17.6%), and new-onset diabetes (14.8%). Jaundice, steatorrhoea, and pancreatitis had the highest positive predictive values (PPV) for pancreatic cancer (1.96%, 1.77%, and 0.89%, respectively). Among the tools, eRAT had the highest PPV of 1.37% (95% confidence interval [CI] = 1.12 to 1.66); the PPV for QPaC was 1.01% (95% CI = 0.82 to 1.22) and QCancer^®^ was 0.8% (95% CI = 0.54 to 1.15).

**Conclusion:**

When applied to Australian primary care data, none of the tools were strongly predictive of pancreatic cancer. New diagnostic models incorporating additional data could potentially improve their predictive performance.

## How this fits in

In the UK, risk assessment tools have been developed and implemented within general practice to detect patients with possible pancreatic cancer based on their symptomatic presentations; the performance of these tools in other healthcare settings is unknown. This study evaluates the diagnostic performance of two UK pancreatic cancer risk assessment tools and an Australian diagnostic algorithm, using linked Australian primary care data. None of the three tools reliably identified patients with underlying pancreatic cancer when applied to Australian primary care electronic medical record (EMR) data. Further research is needed to evaluate whether integrating pancreatic cancer symptoms with additional data recorded in primary care records, such as blood test results and comorbidities, could improve the performance of such tools in this setting.

## Introduction

In Australia, pancreatic cancer was the eighth most commonly diagnosed cancer and third most common cause of cancer deaths in 2024.^1^ Eighty per cent of diagnoses occur at an advanced stage^2^ leading to less favourable prognoses, with a 13% estimated 5-year survival rate.^3^

Patients with pancreatic cancer usually initially present to primary care, therefore considerable efforts have been directed towards enhancing early diagnosis in this setting. Risk assessment tools aim to identify which patients presenting with signs and/or symptoms in primary care are at higher risk of having undiagnosed pancreatic cancer, to prioritise them for further investigation or referral. Some risk assessment tools and diagnostic algorithms have been developed to flag these patients at increased risk such as the electronic Risk Assessment Tool (eRAT),^4,5^ QCancer^®^,^6,7^ and the Queensland Institute of Medical Research (QIMR) Berghofer Pancreatic Cancer Decision Support Tool (QPaC Tool).^8,9^ eRAT and QCancer^®^ were developed using primary care electronic medical records (EMRs) in the UK and have been integrated in GP medical record systems. However, the Australian primary care context presents distinct differences in data recording and healthcare practices, including patient and GP behaviours, use of investigations, and access to specialist care.^10^ This may affect the predictive performance of these tools when applied outside the UK. The QPaC Tool was developed for the primary care setting based on expert clinical input and a modified Delphi consensus process. It comprises a series of guidelines and recommendations that are intended to be used by clinicians during the patient consultation in patients presenting with signs or symptoms of possible pancreatic cancer.

To date, no study has examined the performance of these tools in detecting pancreatic cancer in the Australian setting using routinely collected primary care data to understand their performance and potential clinical utility. This study aims to use linked Australian primary care EMR data to investigate and compare the diagnostic accuracy of the three existing tools for detecting pancreatic cancer.

## Method

### Study design, datasets, and population

A retrospective cohort study was conducted using linked primary care and cancer registry data from Victoria, Australia, to evaluate the predictive performance of eRAT, QPaC, and QCancer^®^ for detecting pancreatic cancer. Primary care data were available through the University of Melbourne Primary Care Audit, Teaching and Research Open Network (Patron) dataset (~6% of Victoria’s GP clinics).^11,12^ Pancreatic cancer (International Classification of Diseases [ICD] 10 code C25) cases were identified via the Victorian Cancer Registry (VCR), with linked data available from 30 January 2008 to 30 June 2022.^13^ The Centre for Victorian Data Linkage (CVDL)^13^ facilitated probabilistic person-level matching using GRHANITE technology for de-identified linkage.^14^ Patients aged 40–100 years with a relevant sign or symptom recorded in Patron between 1 July 2007 and 30 June 2021 were included (a sensitivity analysis was also conducted restricting to a pre-COVID-19 era; that is, 1 July 2007 to 31 December 2019). Patients were excluded if they had a pancreatic cancer diagnosis before entering the study. The entry age of 40 years was selected as pancreatic cancer is very rare in younger patients.^15,16^ Further information on data sources and patient inclusion criteria can be found in the Supplementary Information S1 under dataset and inclusion criteria.

### Symptom mapping

**Figure 1.**
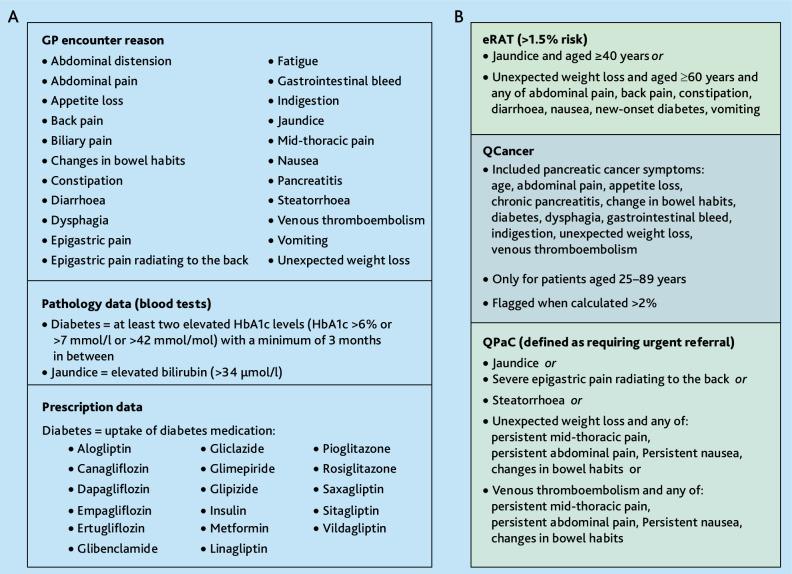
Sign and symptom combination and conditions of the three assessed tools. **(A**) Summary of the Patron data sources used to extract signs and symptoms included in eRAT, QCancer®, and QPaC. All signs and symptoms, apart from diabetes, were mapped using the encounter reason notes, which was provided as free-text data. Several inclusion and exclusion filters were applied to define and extract these signs and symptoms (Supplementary Box S1). Pathology test results (bloods) were used to identify further patients with jaundice based on raised bilirubin levels. New-onset diabetes was defined by either elevated HbA1c levels, two raised results >3 months apart, or through uptake of diabetes medication. Seventeen generic medications, as well as their corresponding brand alternatives (listed in Supplementary Box S1), were included. (**B**) Overview over the sign and symptom combinations and other inclusion criteria used in the three tools included in this study: eRAT, QCancer®, and QPaC. All tools were applied to patients aged 40–100 years. Both eRAT and QPaC have multiple sign and symptom combinations, which would flag a patient as ‘high risk’ of pancreatic cancer. QCancer® calculates risk for multiple cancers, signs and symptoms listed here are those included in the algorithm for pancreatic cancer.

Sign and symptom information in Patron is available using the ‘reason for encounter’ variable, which includes the main reason(s) recorded in primary care by the healthcare provider for each patient consultation. This is equivalent to the ‘problem’ variable in UK general practice EMRs. It can contain multiple signs, symptoms, diagnoses, and administrative queries entered for the same consultation and is primarily recorded as free text. Signs and symptoms were extracted by searching for regular expressions in the ‘reason for encounter’ field, followed by validation by a clinician researcher (see Supplementary Information S1 under symptom mapping and Box S1 for more details). The 23 signs and/or symptoms from the tools included are listed in Figure 1. For QPaC, as it was not possible from our dataset to accurately determine whether patients had persistent symptoms before consulting, the ‘persistent’ criterion were not applied. Additional variables from pathology and prescription data in Patron were used to define diabetes onset^17^ and jaundice^18^ (Figure 1). Detailed inclusion and exclusion criteria are in the Supplementary Box S1

### Tools

Patients meeting eRAT, QCancer^®^, or the updated QPaC criteria^9^ were considered ‘high risk’ for underlying pancreatic cancer and would be considered for further investigation to rule out pancreatic cancer.^^5^^ High-risk status was assigned to those meeting sign and symptom criteria for eRAT (≥1.5% risk), QPaC (defined as requiring urgent referral), or a QCancer^®^ risk score>2% (Figure 1). High-risk sign and symptom combinations included in the eRAT had a PPV >1.5 in the original assessment^4^ (Supplementary Figure S1). The QCancer^®^ tool and code is publicly available on https://qcancer.org, calculating cancer risk for men and women separately (ages 25–89 years) (Supplementary Box S2). Owing to missing data in the Patron registry, body mass index (BMI) and Socio-Economic Indexes for Areas (SEIFA) percentiles were adjusted to standard non-risk figures.

For each tool, the first qualifying sign and symptom marked a patient’s entry to the study. Presence of additional signs and symptoms were extracted for up to 12 months following the first sign or symptom, with data collection ending at the earliest of: patient being flagged as ‘high risk’ by the respective tool (according to the criteria for each tool; Figure 1), pancreatic cancer diagnosis, or 12 months after the first sign or symptom. A 12-month period was selected for this external validation as this was the timeframe used to collect data on sign and symptom combinations for the eRAT and QCancer^®^ studies.^4,7^ The index date was set as either the date a patient was flagged as ‘high risk’ or the last qualifying sign or symptom during this period. Patients were followed up in the VCR for 12 months after the index date to identify any incident pancreatic cancer diagnoses (C25 ICD 10 code).

Detailed description of the tools is available in the Supplementary Information (see sections eRAT, QCancer^®^ *,* and QPaC).

### Statistical methods

Baseline characteristics of the symptomatic study population were compared in patients with and without a subsequent pancreatic cancer diagnosis using Pearson’s χ^2^ and Mann–Whitney U tests. Prevalence of each included sign or symptom (at any point during the study period) was compared in patients with and without a pancreatic cancer diagnosis during the study period. Among patients with a pancreatic cancer diagnosis within 12 months of their index date, the incidence of different signs and symptoms in the 12 months pre-diagnosis was examined.

Diagnostic accuracy statistics (including positive predictive value [PPV], negative predictive value [NPV], positive and negative likelihood ratio, sensitivity, specificity and accuracy, and associated 95% confidence intervals [CIs]) for pancreatic cancer diagnosis within 12 months were calculated in patients flagged as ‘high risk’ by each tool, and for each individual component (sign, symptom, and blood test) of the tools. All analyses were conducted using the statistical programming language R (version 4.3.1). Further details are available in the Supplementary Information.

## Results

### Study population

**Figure 2.**
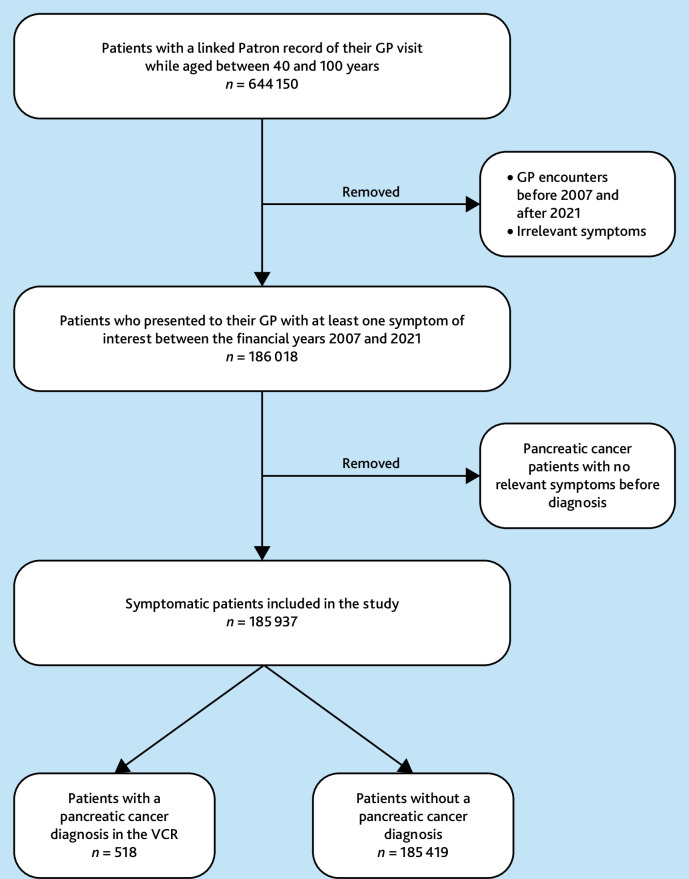
Patient selection. Flowchart illustrating the patient selection process. Only patients with a complete (no missing date or reason for encounter) linked Patron report for encounter reason with a GP were considered. The final symptomatic cohort was identified through limiting the GP encounters to the financial years 2007–2021 to match the timeframe of the available VCR data and avoid exclusion of potential pancreatic cancer diagnoses, as well as excluding encounters with irrelevant symptoms (that is, symptoms not included in the tools) and encounters post-pancreatic cancer diagnosis. VCR = Victorian Cancer Registry.

In total, 644 150 patients aged between 40 years and 100 years had a recorded GP encounter during the study period. Of these, 185 937 patients had at least one presentation with a relevant sign or symptom and were included in this study. Of these, 518 patients had pancreatic cancer diagnosis recorded in the VCR within 12 months of the index date (Figure 2).

Of the total study population, 43.9% (*n* = 81 661) were male and 55.8% (*n* = 103 735) female, with no significant difference in sex distribution when comparing patients with and without a pancreatic cancer diagnosis (*P* = 0.748). Patients with a pancreatic cancer diagnosis had a higher median age at symptomatic presentations (74 years [interquartile range {IQR}: 65, 82] versus 60 years [IQR: 50, 72], *P*<0.001). In the year before pancreatic cancer diagnosis, patients had a median of two GP visits (IQR: 1, 4) (Table 1).

**Table 1.** Patient characteristics

		All patients	Patients with a pancreatic cancer diagnosis	Patients without a pancreatic cancer diagnosis	*P*-value
Total, *N*		185 937	518	185 419	
Sex, *n* (%)	*Male*	81 661 (43.9%)	241 (46.5%)	81 420 (43.9%)	0.748
	*Female*	103 735 (55.8%)	277 (53.5%)	103 458 (55.8%)	
	*Unknown* ^a^	541 (0.3%)	0 (0%)	541 (0.3%)	
Age at presentation, median (IQR)		60 (50, 72)	74 (65, 82)	60 (50, 72)	<0.001
Age at pancreatic cancer diagnosis, median (IQR)			76.4 (66.5, 84.8)		
Number of symptomatic presentations, median (IQR)		4 (2, 9)	4 (2, 7)	4 (2, 9)	<0.001
Number of symptomatic presentations in the year before pancreatic cancer diagnosis, median (IQR)			2 (1, 4)		

Patient characteristics of age, sex, and number of symptomatic visits to the GP for the overall study population and stratified by patients with and without a pancreatic cancer diagnosis. Differences between sexes were assessed using Pearson’s χ^2^ test, for the remaining continuous variables, Mann–Whittney U test was applied. A *P*-value of <0.05 was considered statistically significant. ^a^Patients with sex unknown were not included when assessing statistical differences between sexes.

### Prevalence of individual signs or symptoms

**Figure 3.**
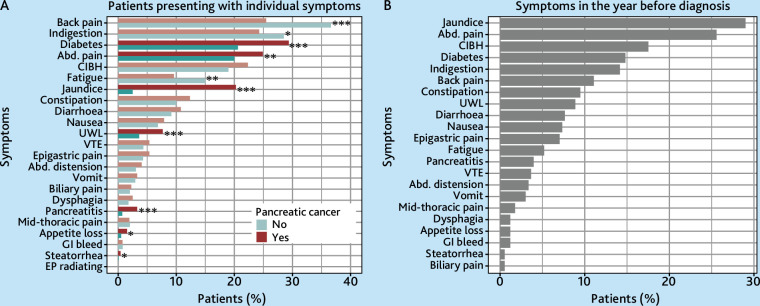
Distribution of signs and symptoms in the study population. Figure showing the proportion of patients with at least one presentation of the listed signs and symptoms. (**A**) Bar chart comparing the proportion of patients with each sign or symptom at any point in the study period in patients with and without a pancreatic cancer diagnosis. Signs and symptoms with a significantly higher proportion in patients with a future pancreatic cancer diagnosis are highlighted in darker shades of colour. Statistical significance was tested using Pearson’s χ^2^, with *P*-values represented as follows: ***<0.001, **<0.01, *<0.05. (**B**) Bar chart illustrating the proportion of patients diagnosed with pancreatic cancer who presented to primary care with at least one sign or symptom of interest in the 12 months before diagnosis (*n* = 324). Abbreviated signs and symptoms: Abd. distension = abdominal distension. Abd. pain = abdominal pain. CIBH = changes in bowel habits. EP radiating = epigastric pain radiating to the back. GI bleed = gastrointestinal bleed. UWL = unexpected weight loss. VTE = venous thromboembolism. Percentages indicate the proportion of patients within each group or cohort who exhibited the respective signs and symptoms.

Patients with a subsequent pancreatic cancer diagnosis had a significantly higher rate of new-onset diabetes (29.3% versus 20.6%, *P*<0.001), presentations with jaundice (20.3% versus 2.5%, *P*<0.001), pancreatitis (3.3% versus 0.7%, *P*<0.001), or unexpected weight loss (7.7% versus 3.6%, *P*<0.001) compared with patients without pancreatic cancer during the whole study period. They also more commonly presented with abdominal pain, appetite loss, and steatorrhoea (Figure 3a), but consulted less frequently with back pain (25.5% versus 36.6%, *P*<0.001), fatigue (9.6% versus 15.0%, *P* = 0.001), and indigestion (24.3% versus 28.5%, *P* = 0.035) (Figure 3a). The detailed estimates in proportional differences, 95% CI, and *P*-values can be accessed in Supplementary Table S1.

Among the 518 patients who were subsequently diagnosed with pancreatic cancer within 12 months of the index date, 324 (62.5%) attended primary care with at least one of the signs or symptoms of interest in the 12 months before their diagnosis. The most common sign or symptom presentation was jaundice in 94 patients (29.0%), followed by abdominal pain (*n* = 83; 25.6%), changes in bowel habits (*n* = 57; 17.6%), and new diagnosis of diabetes (*n* = 48; 14.8%) (Figure 3b).

### Predictive value of individual signs or symptoms and tools

**Figure 4.**
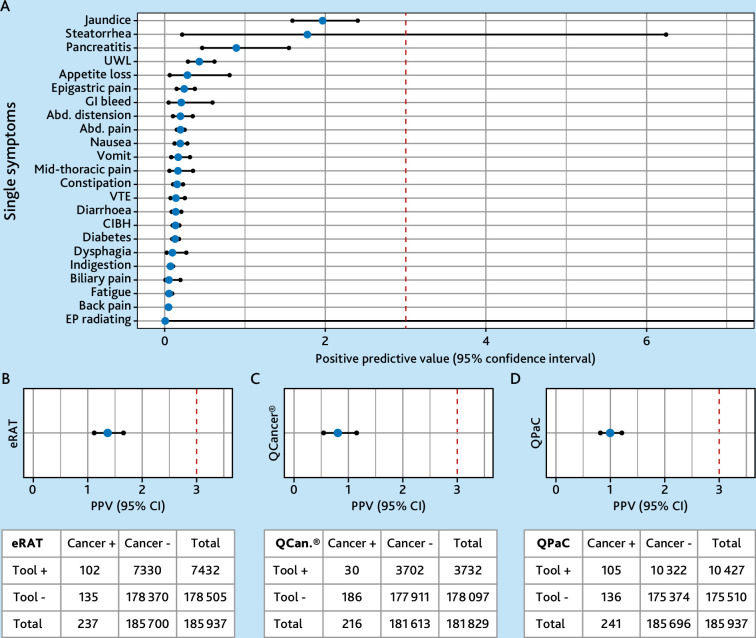
Positive predictive values of the tools and single signs and symptoms. Figure displaying the positive predictive values (PPVs) with 95% confidence intervals (CIs) for (**A**) individual signs and symptoms, (**B**) eRAT, (**C**) QCancer®, and (**D**) QPaC. The PPVs (%) are calculated using the formula (true positives / (true positives+false positives)) * 100. In each graph, the red dotted line at 3% indicates the threshold used in the UK for recommending urgent cancer investigation. Accompanying confusion matrices for each tool show the true and false positives and negatives for identifying patients diagnosed with pancreatic cancer within 12 months of meeting the tool’s criteria (Cancer +/-). The difference in total numbers of patients included in QCancer® is owing to the additional age restriction of the tool. Abbreviated signs and symptoms: Abd. distension = abdominal distension. Abd. pain = abdominal pain. CIBH = changes in bowel habits. EP radiating = epigastric pain radiating to the back. GI bleed = gastrointestinal bleed. UWL = unexpected weight loss. VTE = venous thromboembolism. For presentation purposes, the graph is truncated at 7% PPV with only EP radiating exceeding this cut-off with an upper 95% CI limit of 30.8.

In our cohort, jaundice, steatorrhoea, pancreatitis, and unexpected weight loss had the highest PPVs for pancreatic cancer, at 1.96% (95% CI = 1.58 to 2.4), 1.77% (95% CI = 0.22 to 6.25), 0.89% (95% CI = 0.46 to 1.54), and 0.43% (95% CI = 0.29 to 0.61), respectively, with no other single sign or symptom having a PPV higher than 0.28% (Figure 4a).

After evaluating the predictive performance of each tool for detecting pancreatic cancer diagnosis within 12 months, patients flagged as high risk by the eRAT yielded the highest PPV of 1.37% (95% CI = 1.12 to 1.66). In total, 7432 patients fulfilled the eRAT criteria with 102 patients being correctly flagged as ‘high risk’ and diagnosed with pancreatic cancer within the following 12 months (Figure 4b).

QPaC had a PPV of 1.01% (95% CI = 0.82 to 1.22) with 105 out of the 10 427 patients being correctly flagged as high risk and diagnosed with pancreatic cancer in the following 12 months (Figure 4d).

The QCancer^®^ algorithm flagged 3732 patients with a risk of pancreatic cancer >2%, 30 of whom were consequently diagnosed with pancreatic cancer within 12 months, resulting in a PPV of 0.8% (95% CI = 0.54 to 1.15) (Figure 4c).

Sensitivity analysis showed similar findings when restricting to a pre-COVID-19 timeframe (Supplementary Figure S2 and Supplementary Table S2).

All tools had high negative predictive values of >99.8% and specificity of >94%. The sensitivity of eRAT and QPaC for detecting pancreatic cancer cases was approximately 43%, with QCancer^®^ having a lower sensitivity of 14%. Detailed diagnostic accuracy statistics are presented in Table 2.

**Table 2.** Accuracy statistics for the predictive abilities of the tools

Point estimates (95% CI)	eRAT	QCancer®	QPaC
*Sensitivity (%)*	43.04 (36.65 to 49.61)	13.89 (9.57 to 19.23)	43.57 (37.21 to 50.08)
*Specificity (%)*	96.05 (95.96 to 96.14)	97.96 (97.89 to 98.03)	94.44 (94.34 to 94.55)
*Positive predictive value (%)*	1.37 (1.12 to 1.66)	0.8 (0.54 to 1.15)	1.01 (0.82 to 1.22)
*Negative predictive value (%)*	99.92 (99.91 to 99.94)	99.89 (99.88 to 99.91)	99.92 (99.91 to 99.93)
Positive likelihood ratio	10.9 (9.4 to 12.6)	6.81 (4.88 to 9.51)	7.84 (6.78 to 9.06)
Negative likelihood ratio	0.59 (0.53 to 0.66)	0.88 (0.83 to 0.93)	0.6 (0.53 to 0.67)
*Accuracy (%)*	95.98 (95.89 to 96.07)	97.86 (97.79 to 97.93)	94.37 (94.27 to 94.48)

Accuracy statistics for the predictive abilities of the eRAT, QCancer®, and QPaC to evaluate the effectiveness of each tool in identifying patients at risk of developing pancreatic cancer based on primary care data. Metrics are presented as percentage with 95% confidence intervals where applicable to indicate the reliability of the estimates. Positive and negative likelihood ratios indicate how much a positive or negative flag of the tools increases or decreases the likelihood of a subsequent pancreatic cancer diagnosis.

## Discussion

### Summary

The signs and symptoms most predictive of pancreatic cancer in Australian primary care were jaundice, steatorrhoea, and pancreatitis. The PPVs of the three tools (eRAT, QCancer^®^, and QPaC) remained <1.5%. None of the signs or symptoms or combinations in the three tools exceeded a PPV of 3%, which has been used in the UK as the threshold for considering urgent cancer investigation.^5^

### Strengths and limitations

Use of routinely recorded general practice data represents the setting in which these tools are intended to be used. Information on presenting signs and symptoms was available in the ‘reason for encounter’ notes and signs and symptoms recorded within the broader consultation text could not be captured.^19^ The Patron dataset does not cover all primary care providers in Victoria. Unlike the UK, patients in Australia are not registered to a single practice. Some instances of signs and symptoms may be missed if patients presented at a non-Patron practice resulting in underestimation of PPVs. This effect is likely to be minimal as 90% of Australians visit a regular general practice.^20^

Jaundice was defined based on symptom or pathology result data. Other studies defined jaundice based solely on symptom recording. Including raised bilirubin in the definition^18^ could lower the estimated PPV of jaundice.

The eRAT and QCancer^®^ tools were developed using coded sign and symptom data from UK primary care EMRs. The structure of EMR data in Australia varies considerably from the UK and other settings, with limited use of codes to record signs and symptoms, and greater use of free text to record the main reason for consulting.^21–23^ Variation in clinician recording practices may therefore result in some signs and symptoms being under-recorded in our study.

Greater heterogeneity of symptom recording in Australian practice could potentially contribute to lower PPVs, as could the more recent timeframe examined in this study compared with previous studies.^6,7^ Studies have shown the predictive value of some symptoms has reduced over time as their prevalence in general practice EMRs increases.^24^ The authors had access to cancer registry data up to June 2022 (and therefore sign and symptom data from 12 months before this); future studies could validate our findings using more contemporaneous data as they become available. As this was an external validation, a 12-month timeframe was used to identify sign and symptom combinations, in line with the eRAT and QCancer^®^ development studies.^4,7^ PPVs may be higher if restricting symptom combinations to shorter timeframes, as symptoms occurring within a shorter timeframe may be more strongly indicative of pancreatic cancer than symptoms occurring up to a year apart. Further studies are needed to examine the impact of timeframes.

Victoria is the most urbanised state in Australia and has a lower proportion of First Nation Australians than some states. Therefore, the Patron dataset and population examined in this study may not be representative of general practices and patients across Australia,^12^ and further studies are needed to validate the findings in other Australian states.

Finally, it is possible that better access to general practice in Australia, compared with the UK, means that patients seek help more commonly for the non-specific signs and symptoms of pancreatic cancer. This would also lead to a lower PPV by reducing the prevalence of pancreatic cancer in this symptomatic population.

### Comparison with existing literature

Jaundice is well documented as the most predictive sign or symptom of underlying pancreatic cancer. A study using eRAT reported a PPV for jaundice of 22% in patients aged >60 years.^4^ However, a systematic review of pancreatic cancer symptoms in primary care found a lower PPV of 4.1%.^25^ Both estimates contrast with the 1.96% PPV observed in our cohort. Other symptoms or features examined in the present study have also been previously linked to an increased risk of pancreatic cancer. For example, new-onset diabetes confers a two-fold increased risk,^26^ but was not strongly predictive in our study (PPV = 0.12%), consistent with a systematic review reporting PPVs of 0.07–0.09%.^25^ Similarly, our PPV of 0.43% for unexpected weight-loss, compared with previous reports of PPVs from 0.2–0.8%.^4,7,27^ Our findings are consistent with previous research showing that individual symptoms, such as abdominal pain, back pain, appetite loss, dysphagia, abdominal distension, nausea, vomiting, and changes in bowel habits, do not surpass a 1% PPV for underlying pancreatic cancer.^25^ This study provides further evidence that single signs and symptoms, besides jaundice, do not have the predictive ability to identify patients for urgent investigation for pancreatic cancer in primary care.

The overall predictive performance of eRAT in UK primary care data found a PPV of <3% for weight-loss symptom combinations in patients aged >60 years, increasing to ~22% when using jaundice as a single symptom,^4^ contrasting the lower overall PPV for eRAT of 1.37% found in our study.

QCancer^®^ had an overall PPV of 0.6% for the 10% of patients at highest estimated risk in the original UK study,^7^ and a PPV of 0.2% in an external UK validation study.^27^ Applying a lower 2% PPV threshold to flag patients using QCancer^®^ in our study, a slightly higher PPV of 1.01% was seen in our Australian dataset. It should be noted that QCancer^®^ is the only tool in this study not including jaundice, and PPVs would therefore be expected to be lower than for other tools.

For QPaC no previous studies have examined the predictive performance when applied to EMR data. The present study found that QPaC has similar sensitivity and a slightly lower PPV compared with eRAT when applied across our Australian dataset.

### Implications for research and practice

Our study provides further evidence that individual signs and symptoms have low predictive value for identifying undiagnosed pancreatic cancer in primary care. Furthermore, risk assessment tools combining signs and symptoms did not perform as well as previously reported when applied to Australian data. This is in line with previous external validation studies that have shown that performance of risk prediction tools is often lower when models are applied to populations or settings that are different to those used to build the models.^28^ The QPaC Tool was designed to be used as a desktop or hardcopy guide during GP consultations. When applied as a quasi-electronic decision support tool in the Australian primary care data, its predictive performance for identifying pancreatic cancer was low, which may be owing to incomplete recording of signs and symptoms in the EMR. These tools may therefore perform better if used as guidelines for GPs, rather than tools applied to the patient EMR.

Given the limited performance of existing tools for predicting risk of pancreatic cancer, incorporating information from commonly ordered blood tests, prescriptions, diagnoses, and risk factors could potentially improve the predictive ability of such tools. One study found that 85% of pancreatic cancer cases exhibited elevated fasting HbA1c levels preceding diagnosis, irrespective of their diabetic status.^29,30^ Additionally, abnormal routine blood tests, such as inflammatory markers, are associated with undiagnosed cancer,^22,31,32^ and could potentially enhance risk prediction when combined with other signs and symptoms.^33,34^ Application of machine learning (ML) approaches incorporating temporal patterns of signs and symptoms, disease codes, test results and prescriptions have demonstrated promising PPVs ranging from 32–96%.^35–37^ However, despite their potential, these ‘black-box’ methods still present substantial implementation challenges. These more complex risk prediction models incorporating multiple pre-diagnostic features recorded in general practice EMRs may also experience reduced performance when applied to other countries or healthcare settings compared with the environment where they were developed.^28,36^ Therefore, external validation (as demonstrated in this study), accompanied by retraining and updating of models for the population in which they are intended, is essential.

Applying these tools to populations at higher background risk of pancreatic cancer could also improve their performance. The Enriching New-Onset Diabetes for Pancreatic Cancer (ENDPAC) tool is an example of this, where an algorithm incorporating change in weight, change in blood glucose, and age at diabetes onset is applied to patients aged ≥50 years with a new diagnosis of diabetes.^38^ As these patients have a higher baseline risk of pancreatic cancer within 3 years (1% risk), the PPV of an ENDPAC score ≥3 for predicting pancreatic cancer diagnosis within 3 years was notably higher (2.0–3.6%) than the PPVs reported in our study.^39,40^ Given that ENDPAC is specifically designed for use in people with new-onset diabetes and our focus was on tools for use in the broader patient population, it was not included in our comparison, but future studies could externally validate ENDPAC using Australian primary care data.

In conclusion, this study provided the first assessment of existing risk assessment tools for underlying pancreatic cancer when applied to routinely collected Australian primary care data. None of the three examined tools had good predictive ability for detecting underlying pancreatic cancer in this setting, with PPVs <2%. Further research is needed to identify combinations of clinical features (signs, symptoms, blood tests, prescriptions, diagnoses) recorded in Australian primary care EMRs that could improve the assessment of pancreatic cancer risk in patients presenting with signs and symptoms to primary care.

## Data

The data that support the findings of this study are available from the VCCC Alliance Data Connect. Restrictions apply to the availability of these data, which were used under approvals for this study. Data are available from https://vcccalliance.org.au/our-work/research-and-translation/data-connect/how-to-access-the-hub/.
